# Early Intervention Services Versus Treatment as Usual for First-Episode Psychosis: A Meta-Analysis

**DOI:** 10.1192/bjo.2026.11247

**Published:** 2026-06-30

**Authors:** Baidyanath Ghosh Dastidar, Prathama Guha, Ashik Zaman

**Affiliations:** Calcutta National Medical College, Kolkata, India

## Abstract

**Aims:**

Early intervention services (EIS) for first-episode psychosis (FEP) aim to improve clinical and functional outcomes, yet uncertainty remains regarding their effectiveness compared with treatment as usual (TAU) across heterogeneous service models. This systematic review and meta-analysis evaluated the effectiveness of EIS versus TAU on symptom severity, relapse, hospitalisation and functional recovery in individuals with FEP.

**Methods:**

We conducted a PRISMA-compliant systematic review and meta-analysis prospectively registered with PROSPERO (CRD420251012486). PubMed, Embase, PsycINFO and the Cochrane Library were searched from inception to March 2025 for randomised controlled trials comparing EIS with TAU in individuals experiencing FEP. Two reviewers independently screened studies, extracted data and assessed risk of bias using the Cochrane Risk of Bias Tool, with discrepancies resolved by consensus. Random-effects meta-analyses estimated pooled standardised mean differences (SMDs) and odds ratios (ORs) with 95% confidence intervals. Heterogeneity was assessed using I² statistics. Meta-regression explored moderators including timing of intervention, participant age and inclusion of cognitive-behavioural therapy (CBT).

**Results:**

Twenty randomised controlled trials involving 4,562 participants were included, with follow-up durations ranging from 12 to 24 months. Compared with TAU, EIS was associated with lower symptom severity (PANSS SMD=−0.38, 95% CI −0.50 to −0.26), reduced relapse rates (OR=0.55, 95% CI 0.40–0.75; number needed to treat=8), fewer hospitalisation days (SMD=−0.44) and improved functional outcomes (GAF SMD=0.47) (all p <0.001). Substantial heterogeneity was observed (I²=98.1%). Meta-regression demonstrated stronger effects when EIS was initiated within three months of psychosis onset, among participants younger than 25 years, and in programmes incorporating CBT. No evidence of publication bias was detected.

**Conclusion:**

EIS is associated with superior clinical and functional outcomes compared with TAU in individuals with FEP, despite substantial between-study heterogeneity. Earlier initiation and inclusion of CBT appear to enhance effectiveness. These findings support prioritisation of scalable EIS models within routine psychiatric services, particularly for young people experiencing early psychosis.

